# Habituation of Arctic ground squirrels (*Urocitellus parryii*) to handling and movement during torpor to prevent artificial arousal

**DOI:** 10.3389/fphys.2014.00174

**Published:** 2014-05-09

**Authors:** Sherri L. Christian, Brian T. Rasley, Tanna Roe, Jeanette T. Moore, Michael B. Harris, Kelly L. Drew

**Affiliations:** ^1^Department of Chemistry and Biochemistry, Alaska Basic Neuroscience Program, Institute of Arctic Biology, University of Alaska FairbanksFairbanks, AK, USA; ^2^Department of Biochemistry, Memorial University of NewfoundlandSt. John's, NL, Canada

**Keywords:** habituation, handling and movement, hibernation, *Urocitellus parryii*, Arctic ground squirrel

## Abstract

Hibernation is a unique physiological adaptation characterized by periods of torpor that consist of repeated, reversible, and dramatic reductions of body temperature, metabolism, and blood flow. External and internal triggers can induce arousal from torpor in the hibernator. Studies of hibernating animals often require that animals be handled or moved prior to sampling or euthanasia but this movement can induce changes in the hibernation status of the animal. In fact, it has been demonstrated that movement of animals while they are hibernating is sufficient to induce an artificial arousal, which can detrimentally alter experimental findings obtained from animals assumed to be torpid. Therefore, we assessed a method to induce habituation of torpid hibernators to handling and movement to reduce inadvertent arousals. A platform rocker was used to mimic motion experienced during transfer of an animal and changes in respiratory rate (RR) were used to assess responsiveness of torpid Arctic ground squirrels (AGS, *Urocitellus parryii*). We found that movement alone did not induce a change in RR, however, exposure to handling induced an increase in RR in almost all AGS. This change in RR was markedly reduced with increased exposures, and all AGS exhibited a change in RR ≤ 1 by the end of the study. AGS habituated faster mid-season compared to early in the season, which mirrors other assessments of seasonal variation of torpor depth. However, AGS regained responsiveness when they were not exposed to daily handling. While AGS continued to undergo natural arousals during the study, occurrence of a full arousal was neither necessary for becoming habituated nor detrimental to the time required for habituation. These data suggest that even when torpid, AGS are able to undergo mechanosensory habituation, one of the simplest forms of learning, and provides a reliable way to reduce the sensitivity of torpid animals to handling.

## Introduction

Hibernation is comprised of multiple physiological adaptations used to survive seasonal periods of resource scarcity. Hibernation is characterized by multi-day periods of profound reductions in body temperature [down to -3°C in Arctic ground squirrels (AGS)], metabolic activity (2–4% of resting metabolic rate), heart rate and RR, collectively known as torpor (Barnes, 1989; Carey et al., 2003). These multi-day bouts of torpor are interrupted at regular intervals by brief (8–24 h) periods of arousal throughout the hibernation season where body temperature, metabolism, heart rate, and RR rapidly return to non-hibernating levels. During torpor, EEG becomes isoelectric and animals retain coordinated postures and arouse either spontaneously or in response to external stimulation (Drew et al., 2007).

The metabolic or external triggers that induce natural spontaneous arousal are unclear and are currently under investigation (Jinka et al., 2011, 2012; Olson et al., 2013), but both physical stimuli or changes in temperature can artificially induce arousal (Twente and Twente, 1968). In response to artificial arousals induced by physical manipulations, the first response observed is increased RR followed by shivering which leads to warming of the body beginning at the head, a process that can take up to 3 h (Toien et al., 2001; Weltzin et al., 2006). Since changes to RR occur well in advance to the other physiological changes, changes to RR are commonly used as an early indicator of responsiveness. In addition, the intensity of stimulation needed to induce an artificial arousal varies with both season and time spent in torpor. Specifically, torpid ground squirrels require less stimulation to arouse early and late in the hibernation season compared with mid-season and are more likely to arouse in response to physical stimulation in the last half of each torpor bout (Twente and Twente, 1967, 1968; Harris and Milsom, 1994).

Habituation is an elementary form of learning in which repeated presentation of a stimulus decreases the response to that stimulus. Since torpid animals show no measurable cortical EEG activity (Strijkstra et al., 1999), the ability of a torpid animal to habituate to an external stimuli would suggest that sensitivity to external stimuli is controled by sub-cortical mechanisms. In addition, habituation of a torpid animal to handling has important practical implications for the hibernation researcher since it minimizes the reaction of torpid animals to external stimuli including handling and movement.

Previous evidence for the habituation of the Golden-mantled ground squirrel to movement has been documented (Pengelley and Fisher, 1968), however, this movement consisted of “tossing the squirrels two to three feet in the air,” which is a fairly extreme stimulus. While the squirrels ceased to arouse in response to repeated “tossing,” the stimulus was not quantifiable and has obvious technical disadvantages in terms of reproducibility.

Therefore, we assessed a quantifiable and reproducible method by which AGS could be habituated to a mechanosensory stimuli by analyzing the response of AGS to multiple exposures of handling and/or movement by monitoring changes to their RR, a highly sensitive measure of responsiveness. Our data show that habituation can occur in these animals and that it can occur in the absence of a full arousal, which suggests that coordinated cortical activity is not necessary for habituation.

## Materials and methods

### Animals and husbandry

The Institutional Animal Care and Use Committee of the University of Alaska Fairbanks (UAF) approved all procedures. AGS were trapped on the northern slope of the Brooks Range, Alaska, approximately 32.2 km south of the Toolik Field Station of UAF (68°38′ N, 149°38′ W; elevation 809 m) in July 2004, 2005, and 2006 under permit from Alaska Department of Fish and Game. Upon arrival at UAF, AGS were screened for salmonella and quarantined for 14 days. AGS were housed individually in cages containing white spruce wood shavings (Northland Wood Products, Anchorage, AK, USA) for bedding, and cotton batting as nesting material (Hoch & Selby Co, Portland, OR, USA) at an ambient temperature (T_a_) of approximately 18°C. AGS were fed approximately 40 g of Mazuri Rodent Chow (PMI International) per day. The lighting (48 lx) mimicked the changes in photoperiod at 64° latitude where the light:dark cycle changes from 20 h:4 h to 16 h:8 h over the 14 day period. In early fall, AGS were fed 10–15 sunflower seeds each day for 2 weeks before being moved to 4.57 × 2.90 × 2.1 m environmental chambers (Refrigeration Engineering Co, rebuilt by EJS Systems, Chagrin Falls, OH, USA). with a light:dark cycle of 4 h: 20 h at T_a_ of approximately 2°C and fed rodent chow *ad libitum*. Lights routinely came on at 08:00 h daily. AGS were monitored daily by Animal Quarters staff. Husbandry consisted of periodically changing soiled bedding, feeding and watering each animal, and daily assessment of hibernation status of each animal (as described below). Animals were exposed to animal quarters staff in the chamber for 15–45 min per day, during the 4 h of light. The 4 h of light exposure is greater than wild AGS would experience since they hibernate in underground burrows (Buck and Barnes, 1999), but is necessary to standardize conditions and facilitate husbandry.

The “shavings added” method was used to monitor the hibernation status of AGS. AGS build a cup-like cotton nest so that the back of the animal remains visible during torpor. Shavings are placed on the back of the AGS and checked every 24 h. An AGS was considered to have maintained a torpid state if the shavings were undisturbed upon subsequent observation. Alternatively, animals were determined to have exhibited a spontaneous arousal if the shavings were disturbed or missing. This “shavings added” method is a reliable indicator of full arousals (Lyman, 1948; Pengelley and Fisher, 1961). Additionally, RR and presence of shavings of each AGS were assessed prior to the start of each trial to ensure that AGS were torpid (defined by RR ≤ 5 breaths/min). To minimize disturbance of the animals to light, a head lamp with a red filter (Petzl, Crolles, France) was used when chambers were entered during the dark cycle.

### Habituation trials

To ensure that AGS had entered the hibernation season, only animals that had exhibited at least three torpor bouts, each lasting a minimum of 4 days and punctuated by spontaneous arousal, were randomly placed into an experimental group. The length of each torpor bout typically increases as the season progresses so torpor bout length was typically longer than 4 days once the AGS had reached the third torpor bout of the season. To restrict habituation to the early portion of a bout, which can last 2–3 weeks, the experiments commenced on day 2 through day 5 of a torpor bout with one trial per day.

**Figure 1:**
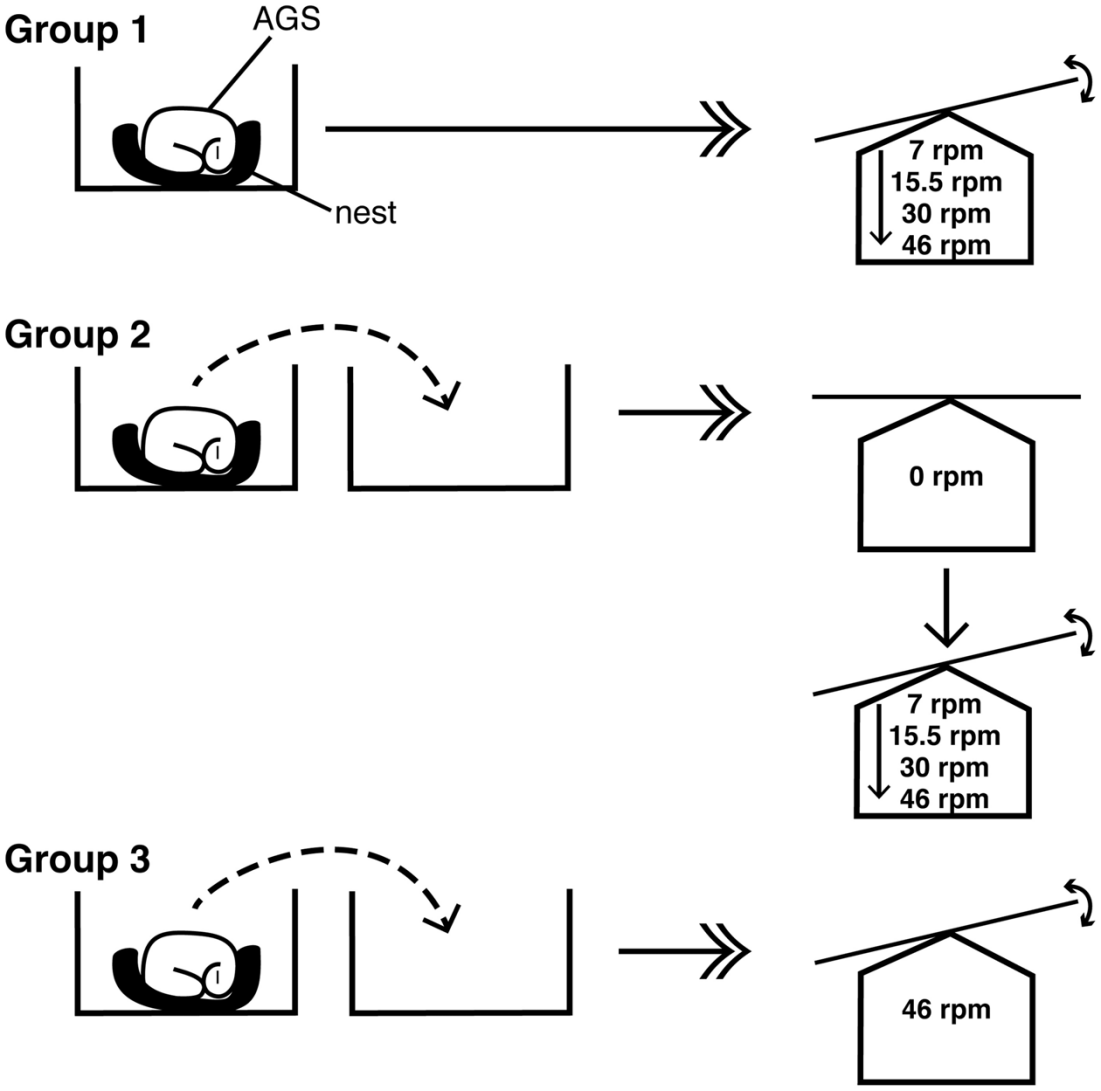
**Schematic diagram of exposure of AGS to handling and movement**. Group 1 AGS were left in their home cage, which was placed on a platform rocker (indicated by double arrowhead), at a rocker rate of 7 rpm (movement indicated by double-headed curved arrow) for 2 min. This was repeated daily until animals had an increase in RR of ≤1 at 5 and 30 min after initiation of movement, for 3 consecutive days (i.e., one session per day). Animals were exposed to increasing rocker rates at frequencies of 15.5, 30, and 46 rpm increasing in a stepwise fashion (solid arrow). Group 2 AGS were handled with the researcher picking them up their cotton cup-like nest and transferring them to a new cage (dashed curved arrow), which was placed on a platform rocker, without any additional movement (i.e., 0 rpm). This was repeated daily as described for group 1 until AGS did not respond to this handling (change in RR ≤ 1) for 3 consecutive days. AGS were then handled as above but the new cage was then placed on the platform rocker for 2 min with movement. The intensity of movement was increased in a stepwise fashion from 7, 15.5, 30 to 46 rpm, once AGS did not respond to the stimuli (RR ≤ 1) for 3 consecutive days. Group 3 AGS were moved with their cotton cup-like nest to a new cage, which was placed on a platform rocker and exposed to a movement intensity of 46 rpm for 2 min. This was repeated daily as described for group 1 until AGS did not respond to handling (change in RR ≤ 1) for 3 consecutive days.

AGS were monitored for responsiveness to handling or movement stimuli by determining the changes in RR, a physiological response that occurs well in advance of an increase in body temperature (Toien et al., 2001). Respirations were directly observed and counted over a period of 1–2 min and recorded as breaths/min. Initial RR was obtained with no or minimal disturbance of the AGS. AGS were then subjected to movement (group 1), or handling with or without movement (groups 2 and 3) as described in detail below and illustrated in Figure 1.

#### Movement in home cage (group 1)

AGS (*n* = 3) were left in their home cage, which was placed on a platform rocker with a tilt angle of ±5 vertical degrees (Model 55S, Reliable Scientific, Nesbit, MS, USA) and set at a rate of 7 rpm for a period of 2 min. The animals were never touched by the researcher, as the entire cage was moved (Figure 1). RR was assessed at 5 and 30 min after initiation of the trial (i.e., 3 or 28 min after completion of movement). The “shavings added” method was then used to assess if a full arousal occurred prior to the next trial. In addition to RR, qualitative assessment of breath type after handling was also assessed to determine if the AGS maintained the shallow, short, low volume, relaxed breath typical of a torpid AGS.

Any increase in RR of a torpid AGS indicates disturbance. To account for error in counting RR we defined an increase of ≤1 breath/min as an indication of habituation and chose to observe this on 3 consecutive days to minimize error associated with the stimulation or counting. Therefore, the movement was repeated daily until the animal exhibited less than a 1 breath/min increase in post-trial RR for three consecutive daily trials, at which point, animals were scored as habituated to this stimulus intensity.

Following habituation to the 7 rpm stimulus, stimulus intensity was increased and the exposure was repeated with rocker speed increased in a stepwise fashion to 15.5, 31, and finally 46 rpm. Data was collected on all animals at each rocker rate for the first 3 days. After this time, any animal that met the criterion of habituation was moved to successively higher rocker rates. Analysis of the total number of days at a specific rocker rate was taken as an indication of the rate for a particular AGS to become habituated.

#### Habituation to handling and gradually increased movement intensity (group 2)

AGS (*n* = 3) were handled by picking up the animal with its cotton cup-like nest without changing the animal's posture or relative horizontal position, to a second plastic cage (Figure 1). Handling lasted for approximately 15 s. The second cage was then placed on the rocking table as above. After the 2 min with or without additional exposure to movement, the animal was returned to its home cage where the post-exposure assessment of RR was performed as above. Animals were assessed for changes in RR in response to handling stimuli alone (i.e., 0 rpm), and then in response to handling plus movement stimuli at 7 rpm with stepwise increases to 15.5, 31, and 46 rpm, as above.

#### Habituation to handling and maximum movement intensity (group 3)

AGS (*n* = 7) were exposed to handling stimuli during transfer to a new plastic cage, as described above (Figure 1). The new cage was then placed on the rocking table and the movement stimuli was kept constant at the maximum rate of 46 rpm. The sample size was increased for this group to ensure an adequate number of animals for analysis because it was unclear if AGS would be capable of habituating to this fairly extreme stimulus.

#### Retention of habituation

Once animals from groups 2 and 3 had reached criterion defined as habituation (an increase of less than 1 breath/min on 3 consecutive days) to handling plus movement at 46 rpm, they were re-tested for habituation to handling and movement stimuli (46 rpm) once per week for 5 weeks commencing 1 week after reaching criterion for habituation. Re-testing for the habituated behavior was only performed after AGS had been in a torpor bout for at least 2 days. On occasion, an AGS would be in an arousal phase or had not been in a hibernation phase for at least 2 days when testing was scheduled and so could not be tested that week.

#### Effect of season on habituation

To assess if AGS would habituate more quickly during the middle of the season than during the fall season, a separate set of animals was tested for habituation to a series of handling only (0 rpm) stimuli trials beginning either October 2nd (early fall; *n* = 4) or January 2nd (mid-winter; *n* = 5).

### Statistical analysis

Statistical analysis was done in R (R Core Team, 2013) unless otherwise indicated. Pairwise comparisons were analyzed by the Wilcoxon rank sum test and correlation determined using Spearman's Rank-Order Correlation test. A linear mixed-effects model (lme) (Lindstrom and Bates, 1990) analysis was used for analysis of trial number, rocker rate, or stimulus effects with individual AGS specified as a random effect. A Chi-square goodness of fit test for the retention of the habituated behavior was performed using JMP9 (SAS Institute Inc. Cary, NC, USA). Data are shown as means ± s.e.m. Significance was set as *P* < 0.05.

## Results

### Habituation to handling and movement

**Figure 2:**
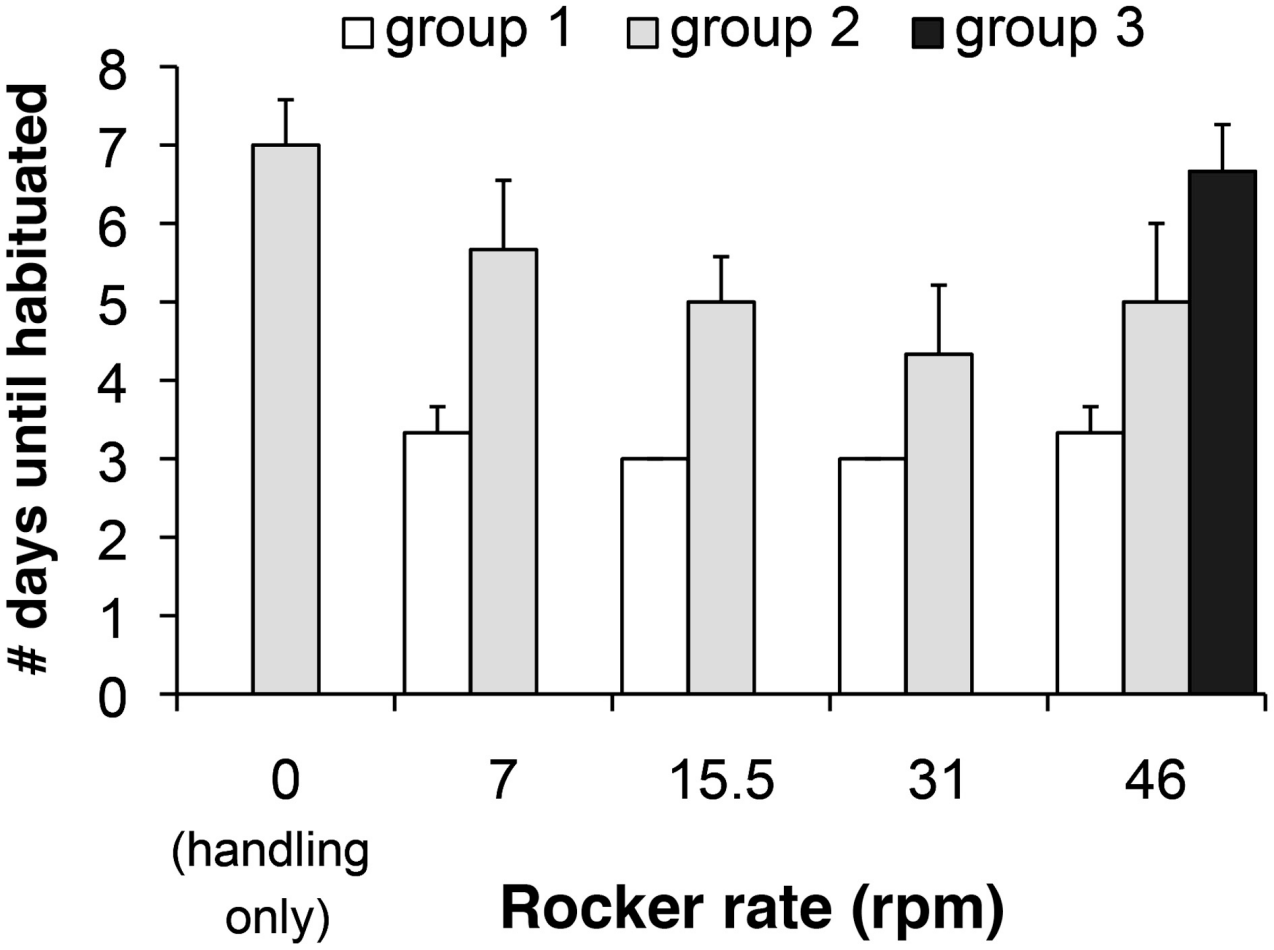
**Hibernating AGS were subjected to handling (being transferred to a new cage) and/or movement as described and the total number of days at the particular stimuli is shown on the y-axis**. AGS that had a change in RR of less than 1 over three consecutive days were considered habituated and the total number of days, including these 3 days, is shown. Rocker rate is shown as rotations per minute (rpm). *n* = 3 AGS groups 1 and 2; *n* = 7 AGS group 3, data are presented as mean ± s.e.m. The statistical analysis is described in the text.

Moving a torpid hibernator from an animal facility to a research laboratory may be achieved by carrying the home cage to the laboratory or by moving the animal to a transport cage and then to the laboratory. We found that animals responded very little to the motion of the cage when not handled (group 1) regardless of rocker rate and thus reached the habituated criterion on average within the minimal possible number of 3 days (Figure 2). A Spearman's rank-order correlation analysis revealed no association between rocker rate and days to habituate [*r*_s_(12) = 0, *P* = 1]. In this group, only one of three AGS showed an increase in RR of greater than 1 breaths/min in one instance when the home cage was transferred to the rocker for the first time. In addition, qualitative assessments of breathing showed that with the exception of the one instance, all AGS continued normal, shallow breathing, characteristic of stable torpor, in response to increasing cage motion.

Handling, by contrast, produced an increase in RR in 8 out of 10 AGS in groups 2 and 3 within the first 3 days of handling and several days of handling was required before the torpid animals habituated to the stimulus. Animals exposed to handling alone habituated within 7.0 ± 0.6 days (Figure 2). Thereafter, we observed a decrease in the number of days required to reach the habituation criteria with increasing rocker rate (group 2), which a Spearman's rank-order correlation analysis revealed to be significant [*r*_s_(15) = −0.53, *P* = 0.041].

When animals were exposed to handling plus the maximum movement rate (group 3), they habituated by 7.1 ± 0.6 days, which was statistically similar by Wilcoxon rank sum test to handling alone [*t*_(8)_ = −0.143, *P* = 0.890]. Therefore, while the addition of handling has a significant effect on the saliency of the stimulus, it appears that habituation to handling or handling plus movement takes a similar time to occur in torpid AGS.

**Figure 3:**
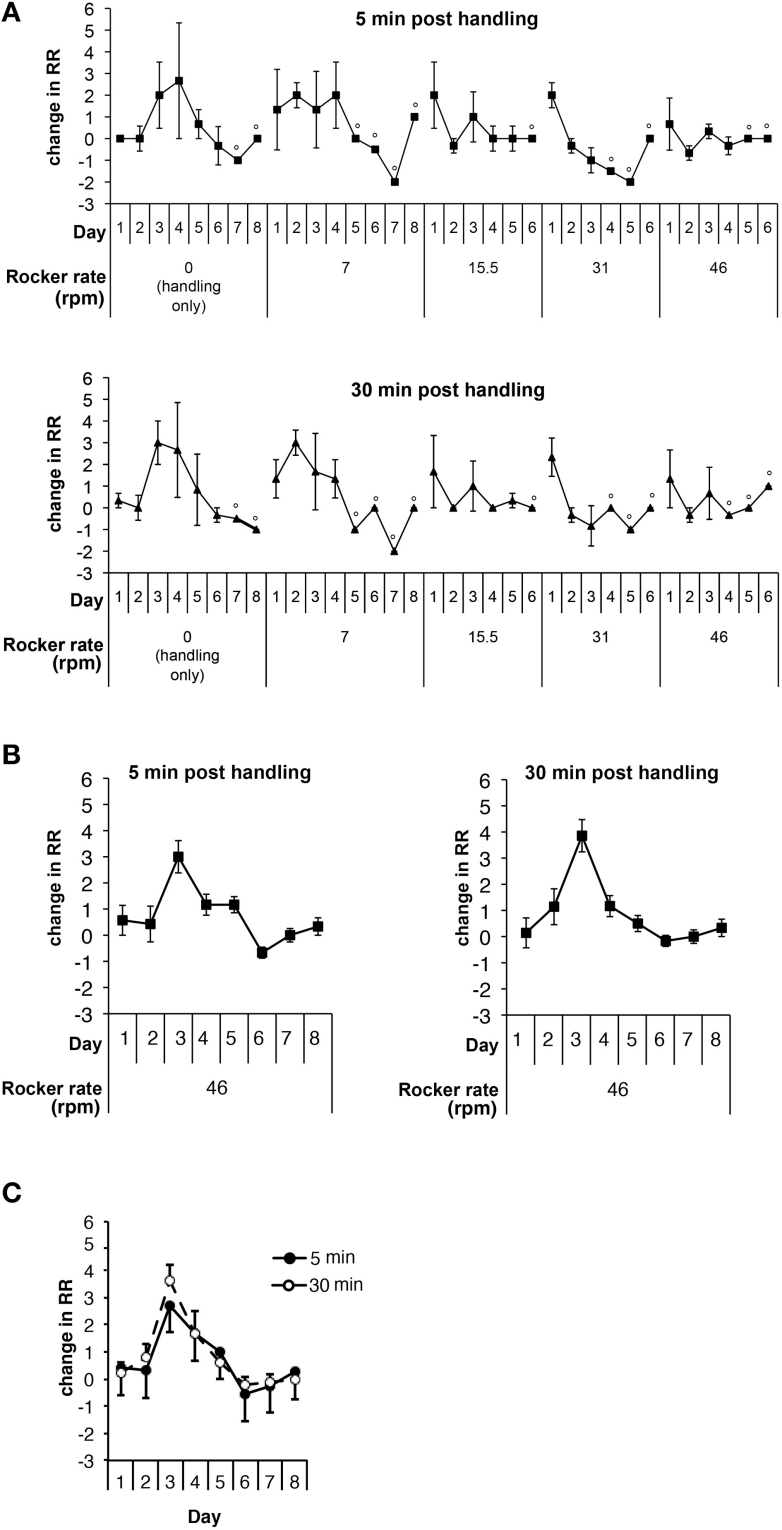
**The change in respiratory rate (RR) of (A) group 2 AGS (*n* = 3) and (B) group 3 AGS (*n* = 7) at 5 or 30 min after handling with or without movement compared to before exposure as measured in breaths per minute**. Rocker rate in rotations per minute (rpm) or without rocking motion (0 rpm, handling only). Change in RR are shown as mean ± s.e.m. One trial was performed per day. “**O**” less than 3 AGS were tested at these times since one or more AGS had progressed to the next level of movement and therefore no error bars are reported. **(C)** Changes in RR in animals from group 2 exposed to handing only and group 3 at 5 or 30 min after handling were averaged (*n* = 10 AGS), data are presented as mean ± s.e.m. A linear-mixed effects model analysis was performed on data shown in **(C)**.

We observed that the change in RR at 5 and 30 min after handling for group 2 AGS subsided with repeated days at each rocker rate (Figure 3A). The initial RR for this group was similar for all trials at 2.1 ± 0.2 breaths/min (handling only), 2.3 ± 0.3 breaths/min (7 rpm), 2.3 ± 0.2 breaths/min (14.4 rpm), 2.5 ± 0.4 breaths/min (31 rpm), and 2.0 ± 0.3 breaths/min (46 rpm), This trend was also seen with group 3 AGS, which were only exposed to one intensity of movement (Figure 3B) and had a initial RR similar to group 2 at 2.1 ± 0.2 breaths/min. In both groups, the largest increase in RR was seen on the third day of exposure, which was followed by a rapid decrease in the change in RR until the last day of assessment.

We therefore assessed if increasing the number of days of exposure had a significant effect on the change in RR of the AGS. We combined the data from group 2 to handling alone and group 3, since both of these groups responded to the stimulus, had not had any previous exposures, and required a similar number of days to habituate (Figure 3C). This allowed us to ensure that there was a minimum of 4 AGS at every exposure day analyzed. Using a linear mixed-effects model analysis, we found that there was a significant decrease in the change in RR as the number of trial days increased [*F*_(1, 125)_ = 7.68, *P* = 0.006] when both 5 and 30 min assessment times were taken into account (Figure 3C) We found no significant difference between assessments at 5 or 30 min [*F*_(1, 125)_ = 0.30, *P* = 0.58].

To determine if assessing animals at either 5 or 30 min after initiating handling or movement would reveal the same effect, we analyzed the change in RR after 5 min using a linear mixed-effects model. This analysis revealed that increasing the number of days trended toward significantly decreased changes in RR after 5 min [*F*_(1, 58)_ = 2.91, *P* = 0.09]. Similarly, we found a trend toward significantly decreased changes in RR after 30 min [*F*_(1, 58)_ = 3.80, *P* = 0.056]. Therefore, while assessments at either 5 or 30 min can indicate the responsiveness of a torpid AGS, combining RR data from assessments at both times will give the researcher the most confidence in determining if an AGS remains responsive.

### Effect of handling and movement on induction of arousals

**Figure 4:**
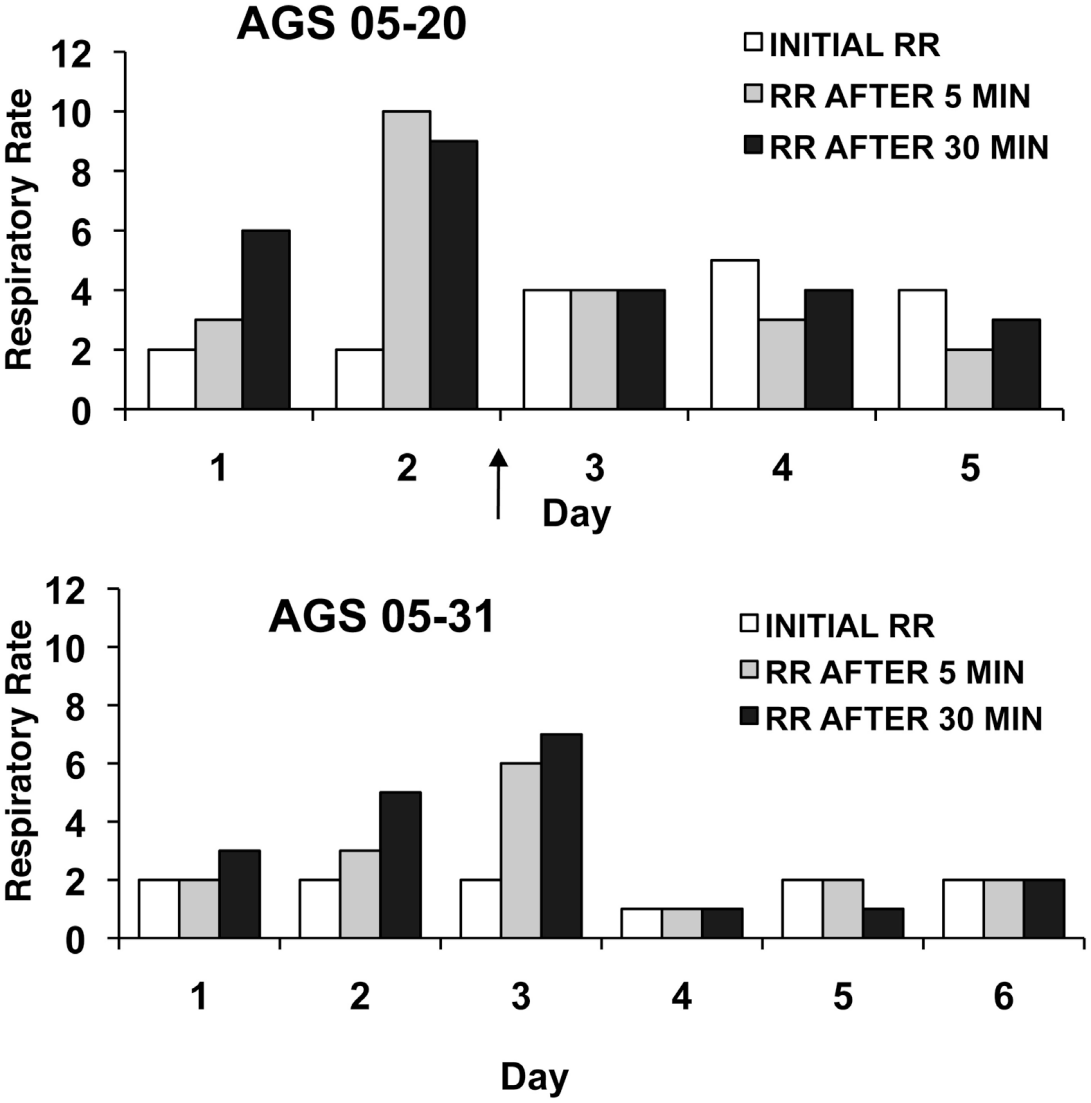
**Example of a respiration pattern from an AGS that habituated immediately after an arousal (top panel) and an AGS that did *not* arouse prior to habituation (bottom panel)**. Respiratory rate (breaths/minute) were measured prior to handling and movement (initial RR) and 5 and 30 min post-handling (RR after 5 or 30 min). **Top panel** shows one animal (identification number: AGS 05-20) over the course of a handling only series of days while the bottom panel shows a different animal (identification number: AGS 05-31) over the course of a handling + movement (7 rpm) series of days. Arrow indicates arousal event.

In addition to measuring changes in RR, the influence of handling and movement on the induction of an arousal was assessed using the “shavings added” method for both groups 2 and 3. The majority of AGS (73%) experienced an arousal after any given trial. However, an increase in stimulus intensity in group 2 induced an arousal only 17% of the time. In 41% of trials an arousal immediately preceded the 3 days of no change in RR to be scored as habituated (see Figure 4 upper panel for an example). This suggests that an arousal event may coincide with habituation. However, in 4 cases AGS habituated without any evidence of a full arousal indicating that an arousal was not necessary for habituation (see Figure 4 lower panel for an example). The presence of an arousal did not significantly affect the time required for habituation to occur. The AGS who aroused prior to habituation took 6.6 ± 0.4 days to habituate (*n* = 8 trials) compared to 6.0 ± 1.2 days (*n* = 4 trials) with no arousal (Wilcoxon rank sum, *Z* = −0.54, *P* = 0.67, *r* = 0.16). Therefore, the occurrence of a full arousal did not appear to be necessary nor detrimental for habituation to occur.

### Retention of habituated behavior

We next tested whether AGS were capable of retaining the habituated behavior in the absence of daily repeated stimuli. We found that animals did not retain the habituated behavior beyond 1 week after the original habituation period (Table 1). Chi-squared analysis showed a significant difference in the frequency of animals that were habituated initially (change in RR < 1 at week 0 post habituation) and the frequency of animals that retained the habituated response at weeks 1–5 post-habituation [*X*^2^, (*N* = 24) = 4.51, *P* < 0.005]. Similar to what we observed with the initial habituation trials, 21% of post-habituation trials induced an arousal.

**Table 1:** **Retention of habituation behavior of Arctic Ground Squirrels (AGS) after habituation to handling and movement**.

**Week post habituation**	**5 min after trial initiated**		**30 min after trial initiated**	
	**Change in RR ≤ 1^a^**	**breaths remained shallow^b^**	**Change in RR ≤ 1^a^**	**breaths remained shallow^b^**
0^c^	5/5	4/5	5/5	4/5
1	1/3	0/3	2/3	0/3
2	3/5	2/5	2/5	1/5
3	2/4	1/4	2/4	1/4
4	2/4	1/4	2/4	1/4
5	1/3	0/3	1/3	1/3

a *The number of AGS that had a change in RR of less than 1 breaths/min after exposure to handling and movement at 46 rpm is shown in the numerator. The total number of AGS tested at each week is shown in the denominator. Five AGS were followed, however, some AGS had aroused spontaneously in the 2 days prior to, or on the day of, assessment and so could not be assessed*.

b *The number of AGS that maintained shallow breathing typical of torpor is shown in the numerator and the total number tested is shown in the denominator*.

c *Week 0 represents the last habituation trial*.

### Effect of season on habituation

Less stimulation has been shown to be required to induce an arousal early in the hibernation season compared with mid-season (Harris and Milsom, 1994), suggesting seasonal changes in the “depth” of torpor with torpor being “deeper” in the mid season than either early or late in the season (Twente and Twente, 1967; Harris and Milsom, 1994). Thus, we assessed the effect of season on the ability of AGS to habituate to handling. Animals that were handled in early fall or mid-winter exhibited similar signs of responsiveness when first handled. However, animals in early fall took 13.5 ± 1.8 days (*n* = 4) with a median, 25th and 75th percentile of 13.5, 11, and 16, to habituate while each of the five animals tested in mid-winter required 4 days to habituate, which was found to be significantly different by the Wilcoxon Rank sum test (*Z* = 2.68, *P* < 0.05, *r* = 0.89). Therefore, the rate of habituation of AGS varies significantly during the hibernation season, with faster rates of habituation in mid-winter compared to fall.

## Discussion

Results reported here suggest that torpid animals can habituate to stimuli that would otherwise induce a response. Once above a minimal intensity of stimulation, we observed that habituation proceeded regardless of stimulus intensity. AGS habituated more quickly during the middle of the hibernation season than in the fall season, which mirrors other assessments of seasonal variation of torpor depth (Twente and Twente, 1967, 1968; Harris and Milsom, 1994). Habituation of AGS did not persist without further stimulus indicating that daily exposure of torpid AGS to stimuli that may cause arousal is required to maintain habituation. Interestingly, occurrence of a full arousal was neither necessary for habituation nor detrimental to the time required for habituation showing that even when torpid, AGS are able to undergo mechanosensory habituation, one of the simplest forms of learning. These data suggest that exposure of torpid animals to stimuli that approximate specific experimental manipulations, or movement necessary to relocate animals, will minimize the influence of experimentally-related stimuli and improve the consistency of data obtained from torpid hibernators.

The finding that AGS habituated to both handling and handling with maximal movement within a similar timeframe suggests that the disturbing quality of the stimuli are not additive. The data suggest that habituation to a stimulus above a minimal threshold requires a similar amount of time regardless of the specific intensity of the stimulus. Thus, there is no advantage to habituating the animals first to handling and then to gradually increasing the intensity of motion. We suggest that initial handling be designed to most closely mimic the disturbance associated with the experimental procedure. Furthermore, observations made after both 5 and 30 min are necessary to determine whether AGS responded to handling, motion and most likely other forms of disturbance. For experimental purposes, the most time efficient approach to habituate a torpid AGS to handling and movement is to expose the animal to both handling and the maximum anticipated movement, with assessment of response after 5 and 30 min.

We also found that torpid AGS can habituate without undergoing a full arousal and that an arousal episode does not interfere with habituation to any given stimulus. Therefore, the occurrence of a natural arousal during a habituation session is not a concern and the AGS can be continuously exposed to the habituation stimuli immediately after an arousal.

Unfortunately, the fully habituated response persists for less than 1 week. Therefore, we recommend that torpid hibernators be habituated to the appropriate stimulus for any given experiment by daily exposure of the torpid animal to that stimulus. Once an animal reaches the criterion used to define habituation, then daily exposure to the stimulus may be continued to ensure that habituation does not dissipate. If experimental manipulations are delayed beyond 1 week, the animal can be habituated again as before.

Finally, it is likely to take more time to habituate a torpid hibernator early in the hibernation season than during the middle of the hibernation season. This suggests that mid-winter AGS may sense the handling stimulus as less intense because they are in a deeper state of torpor, which allows a more rapid rate of habituation. The observation that animals habituate faster during the middle of the hibernation season than during the fall, and thus presumably during the spring, suggests that the effects of handling and other types of stimuli will be easily avoided most readily by habituating torpid animals during mid-season. However, if an extended season is desired, habituating animals throughout the season will minimize artifacts of seasonal differences in the sensitivity of torpid animals to arouse.

Short-term habituation is associated with shorter interstimulus intervals and spontaneous recovery of the response and is distinct from long-term habituation, which is associated with longer interstimulus intervals, and permanent synaptic changes in the brain (Rankin et al., 2009). It is difficult to determine if hibernators experience 24 h as a long or short interval between stimuli since hibernating AGS do not retain daily circadian rhythms (Williams et al., 2012). Based on evidence that some types of habituation require RNA and protein synthesis (Esdin et al., 2010) further research is needed to determine if habituation in hibernating AGS requires protein synthesis given the limitations of protein expression during torpor (Shao et al., 2010). Regardless, any evidence of learning and memory is remarkable at brain temperatures less than 5°C given that both translation and transcription are arrested (Frerichs et al., 1998; Van Breukelen and Martin, 2002) and EEG is isoelectric (Walker et al., 1977; Strijkstra et al., 1999).

Studies that assess the effect of hibernation on physiological and behavioral responses, such as learning and memory (Weltzin et al., 2006), will benefit substantially from habituating torpid animals to handling or movement. Unavoidable physical stimulation is often sufficient to initiate physiological responses that initiate arousal from torpor. Three studies from this lab have already taken advantage of the ability to reduce inadvertent arousals due to handling to ensure that data collected from torpid animals has not been affected by animals responding to handling by the experimenter. These included a study examining the role of mitogen-activated protein kinases in regulating neuronal cell death *in vitro* (Christian et al., 2008), and studies showing the role of adenosine and NMDA receptors in regulating torpor entry and exit, *in vivo*, respectively (Jinka et al., 2011, 2012; Olson et al., 2013).

In conclusion, we have demonstrated and quantified the habituation of AGS to handling and movement during torpor as a practical method to allow hibernation researchers to transport or otherwise handle hibernating AGS without initiating arousal. Changes to RR can be used as a sensitive indicator of responsiveness that is both cost-effective and easy to perform. Habituation should be considered in the design of all studies of hibernating animals.

## Author contributions

Sherri L. Christian, Tanna Roe, Brian T. Rasley and Jeanette T. Moore performed the experiments and analyzed the data. Sherri L. Christian, Michael B. Harris and Kelly L. Drew conceived the study and designed the experiments. Sherri L. Christian, Brian T. Rasley, Michael B. Harris and Kelly L. Drew wrote and edited the manuscript.

### Conflict of interest statement

The authors declare that the research was conducted in the absence of any commercial or financial relationships that could be construed as a potential conflict of interest.

## Abbreviations

AGS: Arctic ground squirrel
T_a_: ambient temperature
RR: respiratory rate
rpm: rotations per minute.
